# “As a patient I do not belong to the clinic, I belong to the community.” Co-developing a multi-level, person-centred tuberculosis stigma intervention in Cape Town, South Africa

**DOI:** 10.21203/rs.3.rs-3921970/v1

**Published:** 2024-02-08

**Authors:** Sally E. Hayward, Nosivuyile Vanqa, Goodman Makanda, Phumeza Tisile, Luthando Ngwatyu, Isabel Foster, Abenathi Mcinziba, Amanda Biewer, Rachel Mbuyamba, Michelle Galloway, Siyavuya Bunyula, Helene-Mari Westhuizen, Jon S. Friedland, Andrew Marino-Medina, Lario Viljoen, Ingrid Schoeman, Graeme Hoddinott, Ruvandhi R. Nathavitharana

**Affiliations:** TB Proof; Stellenbosch University; TB Proof; TB Proof; TB Proof; TB Proof; Stellenbosch University; Beth Israel Deaconess Medical Center, Harvard Medical School; TB Proof; TB Proof; TB Proof; Oxford University; St George’s, University of London; Desmond Tutu Health Foundation; Stellenbosch University; TB Proof; Stellenbosch University; TB Proof

**Keywords:** Tuberculosis, Stigma, Intervention, Cascade of care, Community-engaged research, Human-centred design

## Abstract

**Background:**

Anticipated, internal, and enacted stigma are major barriers to TB care engagement, and directly impact patient well-being. Unfortunately, targeted stigma interventions are lacking. We aimed to co-develop a person-centred stigma intervention with TB-affected community members and health workers in South Africa.

**Methods:**

Using a community-based participatory research approach, we conducted ten group discussions with people diagnosed with TB (past or present), caregivers, and health workers (total n=87) in Khayelitsha, Cape Town. Group discussions were facilitated by TB survivors. Discussion guides explored experiences and drivers of stigma and used human-centred design principles to co-develop solutions. Recordings were transcribed, coded, thematically analysed and then further interpreted using the socio-ecological model.

**Results:**

Intervention components across socio-ecological levels shared common behaviour change strategies, namely education, empowerment, engagement, and innovation. At the individual level, participants recommended counselling to improve TB knowledge and provide ongoing support. TB survivors can guide messaging to nurture stigma resilience by highlighting that TB can affect anyone and is curable, and provide lived experiences of TB to decrease internal stigma. At the interpersonal level, support clubs and family-centred counselling were suggested to dispel TB-related myths and foster support. At the institutional level, health worker stigma reduction training informed by TB survivor perspectives was recommended. Consideration of how integration of TB/HIV care services may exacerbate TB/HIV intersectional stigma and ideas for restructured service delivery models were suggested to decrease anticipated and enacted stigma. At the community level, participants recommended awareness-raising events led by TB survivors, including TB information in school curricula. At the policy level, solutions focused on reducing the visibility generated by a TB diagnosis and resultant stigma in health facilities and shifting tasks to community health workers.

**Conclusions:**

Decreasing TB stigma requires a multi-level approach. Co-developing a person-centred intervention with affected communities is feasible and generates stigma intervention components that are directed and implementable. Such community-informed intervention components should be prioritised by TB programs, including integrated TB/HIV care services.

## INTRODUCTION

Tuberculosis (TB) stigma is a major barrier to ending TB globally.^1^ TB is preventable and curable, yet people with TB, community members, and health workers all report how stigma negatively affects TB care outcomes.^1–6,7^ Stigma delays TB diagnosis^2,8–10^ and prevents treatment completion.^2,11,12^ Anticipated, internal, and enacted stigma^13^ experienced by people with TB are associated with social isolation within families and communities,^14^ and poorer communication between health workers and patients.^15^ TB stigma is also associated with poorer psychological outcomes,^14^ including a higher likelihood of depression.^16^ In high HIV incidence settings like South Africa, TB marks a person for likely HIV co-infection, thus increasing the stigma and discrimination that person experiences.^17^ Intersectional HIV/TB stigma also affects health-seeking behaviour and care outcomes. Specifically, those living with TB and HIV are more likely to miss TB treatment doses if they experienced and felt TB stigma as opposed to those patients who did not experience TB stigma.^11^

Despite global calls to address the wide-ranging impacts of TB stigma,^18,19^ there are few TB stigma reduction interventions and a dearth of high-quality evidence on the design and implementation of such interventions. Two recent scoping reviews on TB stigma interventions reported major gaps in existing literature, including inconsistent application of stigma definitions, variable non-standardised measurement approaches, and incomplete reporting of intervention design and outcomes.^20,21^ Furthermore, the quality of these studies was low, and no studies of psychological interventions measured stigma as a primary outcome.^20,21^ Another review of interventions to address health-related stigma for diseases including TB and HIV highlighted that stigma interventions have hitherto focused on a single socio-ecological level (most often that of the individual), which limits their impact given that stigma drivers and experiences are pervasive across levels including individual, interpersonal, institutional, community, and policy.^22^

To develop effective TB stigma reduction interventions, participatory approaches that put TB-affected communities at the centre of the research process hold great promise. Community-based participatory research (CBPR) involves partnership with community representatives throughout the research process.^23,24^ This attempts to equalise power between researchers and the researched and promotes cultural sensitivity. The aims are to avoid potential harms, maximize the acceptability and reach of interventions, and advance health equity.^25^ This is especially important when developing interventions for marginalised groups, which could include TB-affected communities. In a prior study by our team, community-based stigma assessments conducted by peer research associates demonstrated high levels of anticipated, internal, and enacted stigma that manifests across the cascade of care and identified the lack of quality counselling as a major barrier to care engagement.^26^

In intervention development, co-production is used as a strategy for meaningful collaboration with affected groups in all stages of the design process.^27,28^ Human-centred design (HCD) uses a problem-solving approach that puts the needs of people (service-users) at the centre of the design process.^29^ HCD has been shown to be feasible for intervention development in healthcare settings and across diverse populations,^30–32^ including in efforts to make TB contact-tracing more person-centred in Uganda.^33^ Integrating HCD into CBPR studies can increase the likelihood of intervention effectiveness and adoption, expand its reach, and add innovation.^34^ The aim of this study was to co-develop a counselling-based TB stigma intervention with people affected by TB and health workers using HCD.

## METHODS

### Study design and team

We conducted a qualitative study using a series of group discussions (discussions) to co-develop a counselling-based TB stigma intervention, as part of a community-engaged research study: Use My Voice to EndTB.^26^ This study was co-led by a collaborative team of researchers, including TB survivors from TB Proof, a TB advocacy non-governmental organisation based in South Africa, and the Desmond Tutu TB Centre, Stellenbosch University.

### Study setting and population

This study was conducted in Khayelitsha health sub-district, City of Cape Town Health District, Western Cape Province (WCP), South Africa. The incidence of TB in the City of Cape Town is 462 per 100 000 population.^35^ Khayelitsha is a peri-urban, low-resourced township with a mix of formal and densely populated informal housing and represents one of the highest TB-incidence areas in WCP. We liaised with the TB nurse and the community health workers (CHWs) at Luvuyo, a Primary Health Clinic (PHC) serving the partner community, to identify people with experience of the local TB program by either accessing care, supporting persons with TB, or providing TB services. The community-based group consisted of i) persons with TB (present or past), and ii) caregivers of children or adults with TB. The facility-based group consisted of i) TB counsellors, ii) TB nurses, and iii) CHWs. Individuals were purposively selected to maximize diversity in terms of age, gender, type of TB, and roles (for health workers).

### Data collection processes

Between June 2022 and February 2023, we conducted ten discussions that were approximately ~ 60–90 minutes with a total of 87 participants, which included community-based groups (4 sessions), health worker groups (2 sessions), and subsequently combined community and health worker groups (4 sessions). Discussions were conducted in a venue next to the PHC or in the PHC staff room. Discussions were co-facilitated by a professional nurse researcher (NV), a community-based researcher (LN), and two TB survivors, who were trained as peer research associates (GM, PT),^36^ and have specific experience in qualitative research methods. Discussion guides were informed by the Health Stigma and Discrimination Framework^13^ and pilot tested by the team, with oversight provided by senior researchers (GH and RRN). The discussion guide was designed to cover: 1) Experiences of stigma by domain (internal, anticipated, enacted, and intersectional), 2) Identification of drivers of stigma and how they might impact care at different levels of the care cascade using card decks (described in Use of Human-Centred Design sub-section), 3) Strategies to reduce stigma at different levels of the care cascade, and 4) Potential role of counselling interventions to reduce stigma and key messages that should be delivered. All discussions were audio-recorded, notes were taken by PT and/or NV at all sessions, and summaries of the participants’ responses were written after each session by PT and NV. Discussions were conducted in either *isiXhosa* and/or English in which all data collectors are bilingually fluent.

### Use of Human-Centred Design

HCD has three core phases: inspiration, ideation, and implementation.^29^ This study focused on the inspiration and ideation phases to identify intervention components for future implementation. Building on our prior work,^26^ for the inspiration phase we held six discussions: four with persons with TB and caregivers, and two with health workers. In these, we sought to understand stigma experiences and their drivers, and the perceived needs for stigma reduction interventions. A card deck with visual images of different steps of the TB care cascade, developed by TB Proof for a different community-based project, was used as a prompt to identify scenarios and places where stigma occurs (Appendix). For the ideation phase, we conducted four additional discussions with different combinations of persons with TB, caregivers, and health workers, framed using ‘How might we...’ questions to elicit participants’ suggestions about intervention components and care delivery strategies that could reduce the types of stigma experiences reported in this setting.

### Analysis

We used a thematic approach to analysis.^37^ Specifically, after full immersion in the data from reading and discussing all the transcripts repetitively, two researchers (RRN and SEH) developed a coding scheme inductively with input from NV and GH. This was applied systematically to the data using NVivo 1.7.1 (Lumivero, Colorado, USA), with alterations discussed between the researchers as new codes were iteratively developed. This was complemented by a deductive approach, using the socio-ecological model as a framework for analysis.^38^ The socio-ecological model identifies that factors at multiple levels affect health: 1) individual, 2) interpersonal, 3) institutional, 4) community and 5) policy. We considered how solutions to address stigma mapped to reported stigma experience at each levels and how implementation strategies may have synergies across levels.

### Ethical considerations

This study was approved by the Health Research Ethics Committee of Stellenbosch University (N20/10/113), City of Cape Town Health Directorate and Western Cape Department of Health (NHRD ref: WC_Project ID 9804). All participants provided written informed consent. Community-based group participants received reimbursement for their time and transport (R100 ≈ $5.25), facility-based group participants did not receive reimbursements since the discussions took place in their work setting. Small snacks were provided to all discussion participants.

## RESULTS

Findings are presented according to the levels of the socio-ecological model. For each level, we present key components of interventions proposed by participants to address stigma, which are grouped according to the following behaviour change strategies: education, empowerment, engagement, and innovation. Examples of stigma experiences reported by participants and corresponding stigma intervention components to address these are summarised in Table 1.

### Individual level stigma intervention components

#### Education: optimise existing counselling provided to people with TB by health workers

People with TB emphasised gaps in existing counselling delivered by clinic-based health workers. Counselling, if provided, is focused on treatment adherence, but does not adequately dispel knowledge gaps and misconceptions that can lead to anticipated and internal stigma, including HIV intersectional stigma, as described by a TB survivor:

“If you have TB you are HIV positive in the mind of a lot of people and you end up stigmatizing yourself.” (community participant)

Current approaches to counselling do not address stigma or seek to foster psychosocial wellbeing. A nurse reflected that counselling should address feelings of shame that people with TB may experience and that addressing these psychological effects of TB through counselling could also help health workers better understand and care for people with TB:

“They [people with TB] can be counselled not only to understand what TB is but how they are dealing with shame from themselves of having TB […] I think counselling is very important on how I can deal with things that I come across [as a nurse].” (health worker participant)

To support both well-being and retention in care, people with TB emphasised that counselling must be regular and ongoing throughout care, from first contact with health services to post-TB cure:

“I think when you are diagnosed with TB you must be given counselling regularly when you come to collect your medication.” (community participant)

#### Empowerment: create platforms for TB survivor peer support with guided messaging

People with TB suggested insights from TB survivors could foster stigma resilience, support a sense of self-worth and reduce internal and anticipated stigma, particularly related to fears about death related to TB. They discussed the importance of positive messaging to convey that TB is curable, supported by examples of people who have overcome TB, using illustrative stories of survivors.

“Explain to them clearly so that he can know that he is not the only one who is infected. There are a lot of people who get infected and there is a good chance to be healed, even though it’s not easy. Then you tell them that you are an example of that.” (community participant)

“You always hear that so-and-so had TB and passed away but you don’t hear about so-and-so who survived.” (community participant)

### Interpersonal level stigma intervention components

#### Empowerment: TB support groups to share experiences

Patients and health workers proposed TB support groups for patients to share experiences and support one another through treatment. They also suggested peer navigators are well-placed to lead support groups: to answer questions, share experiences, foster a sense of community, and act as role models.

“We must meet as a group so that you can talk about how you feel... so that I can hear your journey and you can hear mine, so that I can be motivated to move forward.” (community participant)

#### Education: family-centred counselling to improve knowledge, dispel misconceptions and generate support

People with TB and caregivers emphasised the need for family-centred counselling. This creates an opportunity to provide support for caregivers, and allows them to be educated about TB, dispelling myths and reducing stigma. Caregivers could then be better able to support their loved ones in ways that reduce anticipated and enacted stigma due to fear and a lack of awareness about TB.

“You can offer counselling to my family because there is nothing they know about TB.” (community participant)

“Ask the family not to discriminate him/her because it is not the end of the world, he/she will make it.” (community participant)

People with TB and caregivers suggested counselling could take place in homes and communities, outside the health facility, facilitated by CHWs. As such, family-centred counselling could facilitate the provision of patient-centred rather than clinic-centred care, by engaging those affected by TB (not limited to the patient) in a space where they may feel a greater sense of comfort and autonomy.

“Counselling needs to be patient-centred not clinic-centred […] It’s based at the clinic, I as a patient I do not belong to the clinic, I belong to the community. If they can equip these CHWs and add social workers for the community not for the clinic, the stigma can be reduced because the information goes to the community.” (community participant)

### Institutional level stigma intervention components

#### Education: health worker TB stigma training by TB survivors

People with TB proposed training for health workers, led by TB survivors, to improve their understanding of the experiences of people with TB and HIV/TB co-infection and avoid stigmatising language and practices. Health workers also reflected on instances where they or others had dehumanized people with TB and the need to better understand their lived experiences to foster empathy and compassion.

“I am the one who should be advocating for this patient […] but instead it’s me who makes the patient feel less of a human because the patient is infected with TB.” (health worker participant)

“It is a must to listen […] we [health workers] think we know [TB/HIV] but we will never fully know besides what is written in the books.” (health worker participant)

#### Innovation: restructure service delivery models to decrease stigma and improve patient-centred TB care

People with TB and caregivers recommended restructured service delivery models to decrease anticipated and enacted stigma. This includes home-based care, particularly during the first few weeks of treatment, when people with TB are particularly vulnerable and struggle to remain engaged when feeling unwell. Specific suggestions for children with TB included after-school clinic appointments and involvement of school nurses with TB care or to dispel stigma.

“Patients could be met halfway and be given medication at home, because we are not all brave enough in these first two weeks.” (community participant)

“Children should be allowed to come after school [for TB care visits] to prevent questions as to why he didn’t come to school… Each school should also have school nurses to counter issues [that arise due to TB stigma].” (community participant)

Participants also highlight the importance of individualised care, with service delivery models tailored to the patients’ needs.

“Doctors see their patients and they know that they are not the same.. if they could see during that first week that you are having difficulties, how about you get your medication at home for the whole journey? The doctors should look at the situation of the person because we might all be affected with TB but every situation is different.” (community participant)

### Community level stigma intervention components

#### Engagement: increase community awareness and knowledge through community educational forums, school curricula and CHW outreach

People with TB and caregivers proposed solutions to increase awareness of TB in the community, and thus reduce anticipated and enacted stigma. They highlighted the success of previous community events or *imbizos* to raise awareness of TB and HIV, with components such as theatre plays and football matches. They proposed such events should involve TB survivors who can act as role models, encouraging people with symptoms or risk to seek care and those with TB to disclose their diagnosis.

“They brought a big truck to come and educate the community about TB and HIV, there were diverse people, young and old.” (community participant)

“When they put on these plays, they should be played by people who have experience […] for example someone will see [person with TB] whom they know and used to be sick in front of them. They will then think: I know this person and she is a strong woman so […] if she can stand up why can’t I do the same.” (community participant)

Participants mentioned that stigma in the community is mostly due to fear of contagion and lack of knowledge of TB, including gaps in understanding about how effective treatment decreases TB transmission risk or that TB and HIV do not always co-exist.

“You would find out that the person does not know when TB is contagious and when it is not contagious. Even if the person is on the fourth month of the treatment, if it is said that they have TB, people still see them like that [contagious].” (community participant)

People with TB and health workers also proposed incorporating TB into the school curriculum, along with wider outreach by CHWs.

“These outreaches must be done at schools so that [children] can be educated about TB.” (community participant)

“When they do that door-to-door [education], they can reach everyone, and they are able to talk to my family properly about TB.” (community participant)

### Policy level factors

#### Innovation: consideration of how policies impact stigma and how restructured care delivery models including health worker task shifting may help

While policy level components were not explicitly discussed as part of our group discussions, people with TB and health workers identified various issues that can perpetuate stigma in health facilities that would need to be addressed through policy change. They suggested reimagining clinic infrastructure to include removal of specific cards, folders and/or uniforms that could identify care provision to TB patients, and universal mask-use in clinics.

“If we can make it a golden rule that when you come to the health facility everyone must wear a mask because for only them [people with TB] to be asked to wear a mask it’s discriminating, because even someone who didn’t know you will know when seeing you wearing a mask that you are infected with TB and not everyone is comfortable with that being known.” (community participant)

Health workers mentioned high workloads and limited resources, both in terms of personnel and space, impacting time spent with patients, which has direct implications for the provision of quality counselling for people with TB. Health workers also saw the value of home-based counselling with CHW outreach, although they acknowledged the possibility of households being marked by stigma depending on how this was done.

“You have about 50 patients that you have to see in 8 hours.” (health worker participant)

“It would be nice for counselling to be done at home because others are comfortable in their space instead of being told that they should come to the clinic but it mustn’t be too formal where someone will be shocked to see CHWs coming to talk about TB but so that everyone who is at home can know about it.” (community participant)

## DISCUSSION

To decrease TB stigma, action is required at multiple levels. Group discussions with people with TB, caregivers and health workers revealed how stigma is experienced across the socio-ecological model, and guided by HCD principles, participants identified potential intervention components at each level. Intervention components across different levels shared common strategies, namely education, empowerment, engagement, and innovation, which align with intervention functions in the behaviour change wheel framework,^39^ and can facilitate the efficient design of effective interventions. At the individual level, proposed improvements to existing counselling included involving TB survivors, clarifying that not everyone with TB has HIV, emphasising TB is curable and can affect anyone, and providing examples of people who have overcome TB. At the interpersonal level, participants suggested TB support groups and family-centred counselling could dispel myths around TB and foster support. At the institutional level, participants recommended health worker stigma training informed by TB survivors and restructured service delivery models including home-based care. At the community level, approaches to improve knowledge and reduce misinformation included awareness-raising events led by TB survivors, incorporation of TB into school curricula, and increased outreach by CHWs. At the policy level, solutions focused on reducing the visibility generated by a TB diagnosis and resultant stigma in facilities and shifting tasks to CHWs.

These data raise questions about the optimal approach to stigma intervention design that includes intervention components across socio-ecological levels. In a recent systematic review we considered how stigma reduction interventions were implemented at each level of the socio-ecological model.^21^ Existing interventions – all in the pilot stage – typically targeted one or possibly two levels; for example, TB support groups or clubs (individual/interpersonal),^40–42^ home visits (individual/interpersonal),^42,43^ education for patients/caregivers (individual/interpersonal),^44^ health worker training (institutional),^45–47^ and community-volunteer led education (community)^48^. This highlights a tension whereby, given limited resources, trade-offs are made between interventions at different levels of the socio-ecological model. Our participants also identified this friction, with strategies targeting individuals (individuals experience stigma, therefore interventions should seek to foster resilience) often pitted against those targeting the wider family, clinic or, community (interventions should either change attitudes or reduce visibility to prevent stigma from occurring at all). For example, when trying to address hesitation about TB disclosure, some participants argued for structural changes to reduce visibility (removing identifying features in clinics such as cards/uniforms, facilitating home-based care), and others advocated that people with TB should be empowered to freely disclose their TB status (counselling and education to foster resilience).

While single-level interventions may seem more feasible from a funding or policy perspective, a synergistic approach that seeks to foster individual resilience amongst people with TB while strengthening efforts to address community-based stigma is important. This should include providing destigmatized, compassionate health care and dispelling misconceptions about TB that drive community-based stigma. This is consistent with another systematic review of health-related stigma reduction interventions, which commented that effective interventions would be oriented at multiple levels.^49^ Our findings align with other data suggesting peer navigators could improve patient-centred TB education and counselling.^50^ Further, TB survivors can play a critical role in stigma reduction efforts across multiple levels, from providing individual and interpersonal support to informing health worker training and raising community awareness, which may increase the feasibility of implementing multi-level interventions.

The impact of intersectional TB-HIV stigma across all levels of the socio-ecological model, also identified in other studies,^17,51^ was often emphasised by participants. The move towards greater TB-HIV care integration is aligned with the aims of Differentiated Service Delivery (DSD) for HIV treatment.^52,53^ While studies indicate the effectiveness of DSD for TB-HIV when considering indicators such as treatment success,^54^ our findings – in line with other studies^55^ – suggest this could exacerbate intersectional TB-HIV stigma. This has implications for calls to integrate TB with other care services such as diabetes^56,57^ or mental health.^58–60^ Other tensions related to optimal service delivery provision included the challenges of one-size-fits all programmatic approaches. While some patients may appreciate support such as home medication delivery and ongoing counselling throughout treatment, others may find this unhelpful and even condescending. There is a desire for individualised care, tailored to the needs of each person with TB and fostered through empathetic relationships with health workers. Yet resource constraints common in high TB incidence settings were apparent in our data, with health workers reporting how high workloads impact their ability to deliver high quality care. Most literature on individualised care focuses on treatment of chronic conditions in high income countries,^61^ with proposals for tailored TB care largely limited to individualised treatment duration.^62,63^ Implementation research can identify how best to target and deliver individualised care for TB, for example through risk factor screening to identify vulnerable individuals.^64^

Key strengths of our study include the use of HCD and CBPR. Using HCD enabled us to translate stigma experiences and service provision gaps into actionable intervention components. By talking to survivors, caregivers, and health workers in separate and combined groups, we were able to identify differing views and points of synergy. While discussions were conducted in a single clinic in Khayelitsha, Cape Town, which limits generalisability of the findings,^65^ this in-depth focus on a single community is the most appropriate for co-design projects, to form a long-term collaboration with affected groups in a specific community and design an intervention tailored to their specific needs.^66^ Although the use of peer research associates raises the possibility that their lived experience may influence discussions, their involvement improved rapport with participants, understanding of local references, and contextualisation of their narratives.^36^ We thus sought to analyse findings reflexively, bearing in mind the positionality of facilitators and researchers.^67^

## CONCLUSIONS

Decreasing TB stigma requires a multi-level approach. Co-developing a person-centred intervention with affected communities is feasible and can generate directed, tailored stigma intervention components. The actionable intervention components proposed by participants at each level of the socio-ecological framework shared common behaviour change strategies including education, empowerment, engagement, and innovation. TB survivors can play a critical role as peer research associates to both inform stigma intervention design and to help galvanise policy change to decrease factors driving stigma.
